# Big data and artificial intelligence in future patient management. How is it all started? Where are we at now? Quo tendimus?

**DOI:** 10.1515/almed-2020-0014

**Published:** 2020-05-06

**Authors:** Ashraf Mina

**Affiliations:** NSW Health Pathology, Forensic & Analytical Science Service (FASS), Sydney, Australia; Affiliated Senior Clinical Lecturer, Faculty of Medicine and Health, Sydney University, Cameron Building, Macquarie Hospital, Badajoz Road, 2113, North Ryde, NSW, Australia; PO Box 53, North Ryde Mail Centre, North Ryde, 1670, NSW, Australia

**Keywords:** artificial intelligence, big data, deep learning, digital pathology, patient management

## Abstract

**Background:**

This article is focused on the understanding of the key points and their importance and impact on the future of early disease predictive models, accurate and fast diagnosis, patient management, optimise treatment, precision medicine, and allocation of resources through the applications of Big Data (BD) and Artificial Intelligence (AI) in healthcare.

**Content:**

BD and AI processes include learning which is the acquisition of information and rules for using the information, reasoning which is using rules to reach approximate or definite conclusions and self-correction. This can help improve the detection of diseases, rare diseases, toxicity, identifying health system barriers causing under-diagnosis. BD combined with AI, Machine Learning (ML), computing and predictive-modelling, and combinatorics are used to interrogate structured and unstructured data computationally to reveal patterns, trends, potential correlations and relationships between disparate data sources and associations.

**Summary:**

Diagnosis-assisted systems and wearable devices will be part and parcel not only of patient management but also in the prevention and early detection of diseases. Also, Big Data will have an impact on payers, devise makers and pharmaceutical companies. BD and AI, which is the simulation of human intelligence processes, are more diverse and their application in monitoring and diagnosis will only grow bigger, wider and smarter.

**Outlook:**

BD connectivity and AI of diagnosis-assisted systems, wearable devices and smartphones are poised to transform patient and to change the traditional methods for patient management, especially in an era where is an explosion in medical data.

## Background

This article is focused on the understanding of the key points and their importance and impact on the future of early disease predictive models, accurate and fast diagnosis, patient management, optimise treatment, precision medicine, and allocation of resources. Big Data (BD) refers to the fact that data today is often too large and heterogeneous and changes too quickly to be stored, processed, and transformed into value by previous technologies [1], [2]. BD could be found in both structured and unstructured forms. Structured data is the type of data that fits neatly within fixed fields and columns in relational databases and spreadsheets. Relational databases can input, search, and manipulate structured data relatively quickly. The programming language used for managing structured data is called structured query language (SQL). This language was developed by IBM in the early 1970s and is particularly useful for handling relationships in databases [3].

While structured data gives us a birds-eye view of processes in place and outcomes, unstructured data can give us a much deeper understanding of behaviours, associations, trends and intent. The Not only SQL (NoSQL) revolution means that we can find the insight buried within unstructured data without the need to add structure to unstructured data by modelling it for relational data.

Unstructured data cannot be processed and analysed using conventional tools. Examples of unstructured data include text files, medical video data from medical imaging devices (e. g. endoscope, laparoscope, surgery robot, capsule endoscope, emergency video camera, thoracoscope, etc.), biosignal data that have been displayed on the screen of the patient monitor in operating rooms or intensive care units and wearable health monitoring devices, audio data that are verbally or nonverbally created from patients pathophysiologically and medical staffs. Also, mobile activity, social media activity, sensor activity, geolocation activity, satellite imagery, surveillance imagery, and websites. An astonishing 80% of all data generated today is considered unstructured and this number will continue to rise as new internet-connected devices come online [4].

## Introduction

BD and AI promise huge opportunities but raise huge issues. In a report called “Crossing the Quality Chasm”, the Institute of Medicine in the USA identified six aims for improving healthcare quality. These aims are safe, effective, patient-centred, timely, efficient and equitable healthcare. BD resources can be used to improve these six dimensions, used to retrospectively assess the quality of care and to help physicians deliver high-quality care in real-time [5]. Technology integration with the constant seamless flow of data to be captured and analysed real-time require a certain technological platform with certain mix of expertise, which requires substantial change in comparison to the traditional or common structure on many levels including management, IT, talent acquisition, workflow, how outcome is interpreted and its impact on decision making.

BD, AI, and ML will have their application in different areas of health, which will change the way patients are managed. When BD is combined with AI/ML, answers can be found to enable cost reductions, saving time, new product development and optimised offerings, smart decision making, strategic business planning, determining root causes of failures, issues and defects in near-real time, recalculating entire risk portfolios in minutes, and detecting fraudulent or out of norm behaviour [6].

## Major health investments from non-medical organisations

The life sciences will be benefitting from the advances in this area of handling BD achieved by information-power companies like Google, Amazon, Apple, Microsoft and Facebook.

Google is aiming to position itself as a healthcare innovation leader by leveraging its strengths in AI and ML to make sense of vast amounts of health data. Those same types of methods, the infrastructure for managing the data, can all be applied in the health sector. According to the Centre for Medicare and Medicaid Services (CMS), US health spending of 3.3 trillion dollars in 2016 is expected to increase by 5.5% annually on average through 2026 [7]. In fact, with over 7 trillion dollars in health spending per year, it's already almost 10% of global Gross Domestic Product (GDP) [8].

Apple devices and mobile phone Apps allow patients to learn more about their conditions or treatment, doctors can view lab results and radiology images, and nurses can use apps to send and receive secure communications or to help ensure patient safety when administering medications [9]. Apple Watch is poised to detect an irregular heartbeat, e. g. atrial fibrillation, track Parkinson's disease, vision test, hearing test, speech impediments associated with stroke and diabetes control. Also, Apple HealthKit Application Programming Interface (API) is helping the Medisafe app company, which personalise technology to help people better manage their medications, to address the $300 billion drug non-adherence problem.

IBM Watson Health was designed to help health professionals and researchers around the world translate data and knowledge into insights to make more informed decisions about patients care. In oncology, Watson Health is supporting cancer care in more than 300 hospitals and health organisations, and a large, growing body of evidence supports the use of Watson in healthcare [10].

Amazon has formed a team called Grand Challenge which is working on a series of bold projects involving cancer research, medical records, and last-mile delivery. Amazon, JP Morgan Chase Company, and Berkshire Hathaway Company announced the formation of a new healthcare company, providing their employees with technology solutions to access quality care at a reasonable cost, free of profit incentives. There are about 1.2 million employees, combined, scattered across different markets, which means vast amounts of data.

In 2017, Microsoft set up a health department in its Cambridge research laboratory to use AI, ML, and cloud computing to enter healthcare. Patient monitoring solutions, as well as diabetes research, were areas of focus for the tech giant looking to improve healthcare.

In 2017, Facebook filed a patent for an algorithm that attempts to analyse users' emotions by how they type and compare that to their baseline. If people are tapping their phone's keyboard harder or typing slower than usual, that could indicate they are angry or depressed [11].

## Real-world data and real-world evidence

Real-World Data (RWD) is data derived from a number of sources that are associated with outcomes in a heterogeneous patient population in real-world settings [12]. What that means is as long as information on patients such as symptoms, pathology results, radiology, clinical notes, electronic health records, medical claims or billing activities databases, registries, patient-generated data, mobile devices and other relevant information are linked to an outcome such as diagnosis, mortality, recurrence or another outcome, then associations and relationship can be drawn from these data. The integration of genomic data with other RWD sources can enable further stratification of populations leveraging multiple clinical indicators and modelling algorithms [13]. The combination of clinical diagnoses, laboratory test data, and genomic information can be used to identify and stratify patient sub-populations to support biomarker identification, predictive analytics or prospective study development.

Real-World Evidence (RWE) in medicine means evidence obtained from RWD, which are observational data obtained outside the context of randomised controlled trials and generated during routine clinical practice. In order to assess patient outcomes and to ensure that patients get a treatment that is right for them, RWD needs to be utilised. RWE is generated by analysing data which is stored in. It may be derived from retrospective or prospective observational studies and observational registries. In the USA the twenty-first Century Cures Act required the FDA to expand the role of RWE.

RWE comes into play when clinical trials cannot really account for the entire patient population of a particular disease. Patients suffering from comorbidities or belonging to a distant geographic region or age limit who did not participate in any clinical trial may not respond to the treatment in question as expected.

## Big data and pharmaceutical companies

Regulators consider randomised controlled trials (RCTs) as the gold standard for evaluating the safety and effectiveness of medications, but their costs, duration, and limited generalisability have caused some to look for RWE based on data collected outside of RCTs.

Registries and longitudinal healthcare databases, can in some cases replace the need for RCTs. It is worth noting that some studies failed when such databases were used. The key questions to understand why it did not work for regulatory decision making in some cases is linked to two areas of investigation. The first is when we can study drug effects without randomisation, which is controlled by external factors, not by investigators and the other is how to implement a valid RWD analysis to replace RCTs and to avoid mistakes in such analyses [14].

BD can and will accelerate the drug development process by identifying and query cohorts in real-time, analysing co-morbidities and other demographic information without the need for costly patient recruitment, consent forms, and sample sequencing. RWE involves collecting data outside traditional randomised clinical trials and interest in this field is ballooning.

## Big data and rare diseases

Rare diseases are not that rare, 350 million people worldwide have a rare disease, 7,000 rare diseases have been identified, 50% of rare disease sufferers are children, 40% of patients are misdiagnosed initially, an average of 7.3 physicians are seen before diagnosis, an average of 4.8 years before an accurate diagnosis [15].

Patients with rare diseases are not usually diagnosed in a timely manner because the diagnosis can be challenging. As a result, their conditions may well deteriorate before they are diagnosed and be not able to receive effective treatments that could have worked better before they progress to a later stage. BD and AI can predict complex and subtle patterns to diagnose patients and even to predict new unidentified patients. Using RWD on symptomology, diagnoses, treatment history, pathology tests and more in the combination of pattern recognition algorithms to facilitate pattern recognition techniques will help quick diagnosis with a rare disease and identify other patients that are not diagnosed yet.

IMS Health (IMS Health and Quintiles are now known as IQVIA) has RWD portfolio of more than 800 million unidentified patients. They combined RWD and predictive analytics to help detect undiagnosed cases of rare diseases. A team of biostatisticians, data scientists, epidemiologists, and clinical experts applied modern ML methods that incorporate predictive analytics used 70,000 randomly selected patients by an initial algorithm based on risk score, have produced a high-risk group containing 8% of confirmed cases. Then, it was risk scored by another ML algorithm which produced a prevalence of the confirmed diagnosis of 20.5%. But only 0.7% of patients in the sample group had the disease. That study provided evidence that the use of an algorithm could increase the chances of potentially finding high-risk patients earlier [16].

## Big data identifying health barriers causing under-diagnosis

In another study, IQVIA developed a cohort selection algorithm that used Hospital Episode Statistics data covering outpatient, inpatient and Accident and Emergency activity over more than 5 years in England. This revealed a high number of events for 3 years ahead of a formal diagnosis, with over 90% of patients being known to the hospital system within the three-year time frame, and a wide variety in the types of diagnostic pathways to reach a tertiary centre initially. Furthermore, the study identified substantial variability in the incidence rate per 100,000 population suggesting challenges of under-diagnosis in some tertiary centres [16].

## Wearables devices and smartphones role in patient management

Development of consumer wearable devices or sensors is on the increase and profitable area for device makers. Wearable devices, when bought by many people is a better business model that is going to generate more revenue vs. selling a few medical systems in comparison.

Wearable devices, when introduced a few years ago, were in the recreational-grade state, they're changing incredibly rapidly into research-grade and ultimately to clinical grade [17], [18], [19], [20], [21], [22]. Some examples will be given to shed some light on this area. Wrist-worn wearable devices such as Apple Watch Series 2, Samsung Galaxy Gear S3, and Fitbit Charge 2 accurately measure baseline and induced supraventricular tachyarrhythmia heart rate [19]. Also, glucose monitors, which are approved by the Food and Drug Administration (FDA) in the USA, individuals can wear and interface with digital apps, which then connect directly with healthcare providers. In years to come, more health information will exist more outside the health system than inside the health system. Adamant Technologies has created a computer chip that can take the sense of smell and taste and digitise them. That means smartphones, computers or devices can smell for itself. Possible applications are metabolic tracking, monitoring medical conditions like asthma, diabetes, test blood alcohol and even detecting cancer. Korea Advanced Institute of Science and Technology (KAIST) developing a quick and efficient way to diagnose diseases like diabetes or lung cancer. The device uses a highly-sensitive exhaled breath sensor which can be mounted on a smartphone. Made of tin dioxide nanofibers coated with catalytic platinum nanoparticles, the sensor can detect the presence of acetone (a diabetes signal) or toluene (a lung cancer signal) even at concentrations of less than 100 parts per billion. Another example is Cycardia Health has developed a smart bra iTBra for monthly breast scanning. Every 3 min one woman is diagnosed with breast cancer.

## Summary

Because the concept of BD and its application is still in early stages in the healthcare sector, the use of the underutilised data and the need to better understand many diseases will help improve the quality of care. Additionally, the increasing usage and the availability of digital, such as data electronic health records, clinical trial data, e-health applications, genomic, transcriptomic, proteomic, metabolomic and microbiomic data, will leverage BD applications in improving the cost-effectiveness of care, predictive models of disease course and response to therapy, characterisation of disease heterogeneity, drug safety and development, and precision medicine [23], [24], [25], [26]. BD has the potential to improve medical care and reduce costs, both by individualising medicine and bringing together multiple sources of data about individual patients.

The more BD is used in medical science and different sophisticated algorithms being built for causality analysis, the more specific models will be designed according to the requirements of each application [27]. As an example, BD has been used in transfusion for detection of transfusion-related complications, determining patterns of blood usage, identifying trends in blood order schedules for surgery, and in benchmarking. Additionally, BD can monitor compliance with key performance indicators for overall blood inventory and management to optimise the usage of blood [28].

Research from International Data Corporation (IDC), a global market research agency, states that companies with the right data will see an additional $430 billion in productivity gains by 2020. It's no wonder why IBM estimates there will be roughly 2.72 million data science jobs posted over the next few years [29]. About a third of the world's data is generated from the healthcare industry. BD has V6 Characteristics; value, volume, velocity, variety, veracity, and variability are important factors to be considered. BD analytics in medicine and healthcare covers integration and analysis of a large amount of complex heterogeneous data such as various – omics data (genomics, epigenomics, transcriptomics, proteomics, metabolomics, interactomics, pharmacogenomics, diseasomics), biomedical data and electronic health records data [24].

AI, ML and Deep Learning (DL) areas have garnered a lot of attention over the past 2 years. ML is a subset of AI involved with the creation of algorithms which can modify itself without human intervention to produce desired output by feeding itself through structured data. DL is a subset of ML where algorithms are created and function similar to those in ML, but there are numerous layers of these algorithms- each providing a different interpretation to the data it feeds on. Such a network of algorithms are called artificial neural networks, being named so as their functioning is an inspiration at imitating the function of the human neural networks present in the brain [30].

Deep convolutional nets have brought about breakthroughs in processing images, video, speech and audio, whereas recurrent nets have shone a light on sequential data such as text and speech. DL allows computational models that are composed of multiple processing layers to learn representations of data with multiple levels of abstraction. These methods have dramatically improved the state-of-the-art in speech recognition, visual object recognition, object detection and many other domains such as drug discovery and genomics. DL discovers intricate structure in large data sets by using the backpropagation algorithm to indicate how a machine should change its internal parameters that are used to compute the representation in each layer from the representation in the previous layer. DL requires much more data than a traditional ML algorithm. The reason for this being that it is only able to identify concepts and differences within layers of neural networks when exposed to over a million data points. ML algorithms, on the other hand, are able to learn through pre-programmed defined criteria [2].

Applying ML concept in computational pathology, which is defined as an approach to diagnosis that incorporates multiple sources of data (e. g. pathology, radiology, clinical, molecular and laboratory operations), presents clinically actionable knowledge to doctors, investigators and patients [31]. The work culture will play a significant role to be more inclusive to allow different experts from all relent areas to work cohesively together including, IT application specialists, medical and scientific experts, data scientists, hardware and software engineers, biostatisticians, and algorithm designers.

By 2020, information about the body, health, and healthcare is predicted to double every 73 days. In fact, to keep up with Primary Care literature, a general practitioner would need to read for 21 h every day. It has been suggested that integrating technology such as Clinical Decision Support Systems (CDSS) are the only way medical professionals can hope to keep up with the increase in information [32]. As a result, some issues were raised such as not all physicians are in support of the changes and not feeling comfortable that medical decisions are made by using sophisticated models using BD and AI rather than medical expertise only.

From 2011 to 2016, misdiagnosis and surgical complications in public hospitals have cost New South Wales state in Australia, more than $262 million [33]. Cos can be minimised through better preventative measures, better-targeted therapies and increased compliance for medication. Intervening sooner in the course of a patients' health, before they slide into a disease state will save money on unexpected hospitalisations, emergency room visits, and physician visits.

## Outlook

BD has also evolutionary and revolutionary implications in Epidemiology for identifying and intervening on the determinants of population health [34]. BD revolution will vastly improve the granularity and timeliness of available epidemiological information, with hybrid systems augmenting rather than supplanting traditional surveillance systems, and better prospects for accurate infectious diseases models and forecasts. In psychiatry, analysis of the BD will provide unprecedented opportunities for exploration, descriptive observation, hypothesis generation, and prediction, and the results of BD studies will be incorporated into clinical practice. Technical challenges remain in the quality, analysis and management of BD [35]. Worldwide, looking at the bigger picture, there may be a need to establish an international organisation to set the standard and the basis for data sharing, storage, ethics, privacy and security. Heading for future, medical and science schools should expand beyond teaching statistics only and integrate into their curriculum AI, ML, DL, computing and predictive-modelling, and combinatorics.

Health apps and wearable devices are sufficiently known and used and are considered potential supports for greater involvement in health management. The involvement of patients and doctors would be desirable to overcome barriers and boost awareness about privacy and the confidentiality of data [18], [36]. The surge of public disease and drug-related data availability has facilitated the application of computational methodologies to transform drug discovery. Various resources and tools could leverage in order to perform such analyses [37].

The privacy issue is a big concern regarding data sharing, but with the right technology for security in place and masking patients personal information, that should not be a limitation [38]. The same security issue applies to current data and having a bigger pool of data should not be dealt with any different. General Data Protection Regulation (GDPR) is a regulation in the European Union (EU) law on data protection and privacy for all individuals. As BD become increasingly mainstream, it will be important to maintain public confidence by safeguarding data security, governance and confidentiality [39]. Because human beings are living longer than ever before and they want to contribute more to decisions about how and where they receive care. There are growing expectations for better experiences not just outcomes, prevention not only treatment and more personalised care closer to home. The advances in data analytics will help influence community behaviour in priority areas such as childhood obesity, diabetes, antimicrobial resistance, rare disease diagnosis, predictive models and help in identifying and solve crimes. The aspects discussed in this article are not only a leap in technology applied in healthcare but rather interruptive technology that will change the way that the healthcare services is designed and delivered.
